# Extensive striated muscle damage in a rat model of Duchenne muscular dystrophy with *Dmd* exons 10–17 duplication

**DOI:** 10.1186/s13395-025-00386-2

**Published:** 2025-06-09

**Authors:** Jean-Daniel Masson, Valentina Taglietti, François Ruby, Hiroya Ono, Nadir Mouri, Alan Jorge, Laurent Guillaud, Laurent Tiret, Frederic Relaix

**Affiliations:** 1 Univ Paris-Est Créteil, INSERM, U955 IMRB, Créteil, F-94010 France; 2 École nationale vétérinaire d’Alfort, U955 IMRB, Maisons-Alfort, F-94700 France; 3 Département de Biochimie-Pharmacologie, AP-HP, Hôpitaux Universitaires Henri Mondor, Créteil, F-94010 France; 4 EFS, U955 IMRB, Créteil, F-94010 France; 5 AP-HP, Hôpitaux Universitaires Henri Mondor, Service d’histologie, Créteil, F- 94010 France

**Keywords:** Congenital myopathy, Neuromuscular disorder, Inter-individual data correction, Myonecrosis, MYOM3, hs-cTnT, Plethysmography, ECG, Notched T wave

## Abstract

**Background:**

Duchenne muscular dystrophy (DMD) mainly affects young boys with out-of-frame mutations in the *DMD* gene, leading to dystrophin deficiency. This loss disrupts the assembly of the sarcolemmal dystrophin-associated glycoprotein complex, resulting in membrane fragility and damage during muscle contraction-relaxation cycles. Consequently, patients experience progressive muscle weakness, loss of ambulation and cardiorespiratory failure. Gene therapy represents one of the most promising therapeutic approaches, requiring rigorous preclinical validation of candidate strategies. While several preclinical models of dystrophin deficiency mimic point mutations or exon deletions, no existing rat model accurately replicates *DMD* gene duplications, which account for approximately 10% of DMD cases.

**Methods:**

Using CRISPR/Cas9 genome editing, we generated a ~ 125 kbp duplication encompassing exons 10–17 of the *Dmd* gene in Sprague Dawley rats. To characterise disease progression in these rats, we assessed biochemical, histological and functional biomarkers at 6 and 10 months of age, comparing them to their healthy littermates.

**Results:**

We established the R-DMDdup10-17 line. The microstructure of limb, diaphragm and cardiac muscles of R-DMDdup10-17 (DMD) rats exhibited dystrophic changes at 6 and 10 months, including loss of myofibres and fibrosis. These alterations led to a significant body mass reduction, muscle weakness (including diaphragm deficiency) and cardiac electrical defects. Premature lethality was observed between 10 and 13 months.

**Conclusion:**

Duplication of the *Dmd* genomic region encompassing exons 10 to 17 in rats results in dystrophin deficiency, severe striated muscle dystrophy, and premature death. The R-DMDdup10-17 line represents the first reported genetic model of a severe and early lethal duplication variant in the *Dmd* gene. It provides a critical tool for assessing targeted gene therapies aimed to correct such mutations.

**Supplementary Information:**

The online version contains supplementary material available at 10.1186/s13395-025-00386-2.

## Background

Duchenne muscular dystrophy (DMD) is a progressive and degenerative disease characterised by muscle wasting and detrimental remodelling, associated with cardiorespiratory abnormalities, ultimately leading to the death of most patients around the third/fourth decade of life. DMD is a genetic disorder caused by more than 7,000 identified mutations in the *DMD* gene [1] encoding dystrophin proteins. In the striated muscle, the full-length isoform of dystrophin is part of a large glycoprotein complex connecting the cytoskeleton of the myofibre with the extracellular matrix [2]. Among the genetic rearrangements observed in DMD patients, deletions are the most common, accounting for ~ 70% of cases, and primarily occurring in a major hotspot region spanning exons 44–55 [3]. Duplications are less frequent, representing about 11% of cases, and are mainly found in a minor hotspot 5’ region spanning exons 2–12 and 17 [3]. Both deletions and duplications typically result in out-of-frame mutations with premature stop codons leading to absence of dystrophin, which through destabilization of the dystrophin-associated glycoprotein complex ultimately causes muscle fiber damage, myonecrosis and progressive loss of skeletal and heart muscle function [4].

Several animal models of DMD have been reported such as mice, rabbits, dogs, pigs and monkeys [5–9]. Large animal models suffer from individual variability and are expensive and difficult to handle. On the other hand, mice only partially recapitulate the severity of the human disease trajectory, exhibiting mild pathological phenotype, late cardiac impairment, limited muscular lesion and muscle weakness [7, 10]. To overcome these issues, rats models have been generated, by inducing deletions into the minor [11–13] or the major [14] hotspot. However, none of these rat models carries a representative duplication rearrangement in the minor hotspot, limiting the preclinical evaluation of targeted gene therapy approaches.

Here, we report the generation of a novel DMD rat model with a CRISPR/Cas9-induced duplication of exons 10 to 17 of the *Dmd* gene, which we named the R-DMDdup10-17 line. We show that R-DMDdup10-17 rats display reduced life span with premature lethality between 10 and 13 months of age; severe dystrophic remodelling occurs in the heart and skeletal muscles. This pathology closely mimics the progression and severity of human DMD, characterised by prominent myonecrosis, muscle weakness, fibrosis and early cardiorespiratory impairment. We additionally report a well-adapted method of data correction using the cubic tibia length (TL^3^) to eliminate the interference of differential animal growth in DMD conditions.

## Methods

### Rat colony establishment, animal care and use

The R-DMD-dup10-17 rat model was generated by producing a duplication of exons 10 to 17 of the Dystrophin (*Dmd*) gene. Rat exonic and intronic sequences were selected (ENSRNOG00000035692.6) and CRISPOR algorithm [15] was used to identify guide RNA sequences (sgRNAs) for CRISPR mediated genome editing. A ~ 125 kb sequence encompassing exons 10 to 17 was duplicated using two 5’ sgRNAs in intron 9 at > 41 kb from exon 10: ATAAACCCAATGAACCATGC and TATATTCCTGCATGGTTCAT, combined with two 3’ sgRNAs in intron 17 at > 15.5 kb from exon 17: GAAGGAGCATTGGGGCATTA and ATAATGCTGGGAAGGAGCAT. The sgRNA number refers to the MIT specificity score (http://crispor.tefor.net/crispor.py). After duplication, splicing from splice donor of exon 17 to splice acceptor of the duplicated exon 10 was expected to produce an out-of-frame mutation leading to a premature stop codon in the duplicated exon 10. Fertilised oocytes from the Sprague Dawley strain (RjHan: SD provided by Janvier Labs, France) were electroporated with RNP dual RNA (6 µM Cas9 protein, and 8 µM of each sgRNA). Electroporated fertilised oocytes were reimplanted in foster females to obtain animals that were screened by junction genotyping. The duplication junction and two copies of the wildtype 10–17 region were confirmed in the F0-12235 male founder rat. Genotyping was performed on DNA extracted from ear biopsies. PCR mix was prepared with Phusion Taq buffer (Invitrogen), 0.25 mM dNTP, 0.6 µM of the common Ex10-r reverse primer 5’-ATAAGTATTAGGCCATTGTTCAAGG-3’ and 0.3 µM of each of the Int9-f forward primer 5’-ACCCAATGAACCATGCAGGA-3’ for amplifying the wild-type (WT) allele and the Ex17-f primer 5’-TGTTGCTCATTCACTATATGTATGG-3’ for amplifying the R-DMDdup10-17 (DMD) allele, 0.03 U/µl Phusion Taq and around 150 ng of DNA. The PCR was performed with the following program: initial denaturation at 95 °C for 3 min, 33 cycles with denaturation step at 94 °C for 15 s, annealing at 56 °C for 45 s and extension at 72 °C for 10 s, ending with a unique final extension at 72 °C for 3 min. Duplication of exons 10 to 17 (277 bp) was detected on a 2% agarose gel.

Rats were housed in a pathogen-free facility with 12 h light/dark cycles in accordance with the 2021/63/EU European Directive. Only male rats were used for the experiments. Both wild-type and DMD rats were born from the same litters. All the procedures including animal handling were validated by the local ethic committee and approved by the French ministry of Research (APAFIS#44858-2023091920248268). The general condition of the rats was checked daily to record the onset of physical deterioration, which usually occurred around 10 months of age. Limits points were established and constantly evaluated by daily observing the animals individually, including weekends and public holidays. Should the limit points be exceeded, corrective action was taken, and in the event of no benefit (irreversible progression of the disease), we proceeded to compassionate euthanasia of the animals.

### Functional assessment of animals

#### Grip test

Forelimb grip strength was assessed using a grip strength meter (BIO-GS3, Bioseb). Animals were positioned to grasp the T-bar with their forelimbs, then pulled backwards until they released the grip. A total of five repetitions were recorded, with a minimum of 10 min of rest in the rearing cage between two consecutive trials. The maximal force of each rat was corrected for individual TL^3^ following the methodology described above, while the force maintenance index (FMI) was calculated as the average of the last three measurements expressed as a percentage of the maximal force.

#### In vivo muscle electrophysiology

An in vivo muscle contraction test was performed using the 1300 A 3-in‐1 Whole Animal System (Aurora Scientific, Aurora, ON, Canada) and analysed using the dynamic muscle control/analysis (DMC/DMA) high‐throughput software suite provided by the manufacturer. Rats were weighed and placed under moderate anaesthesia with a mixture of 0.03 mg/kg Bupaq (Virbac), 0.2 mg/kg Sedator (Dechra) and 50 mg/kg Ketamine 1000 (Virbac) administered subcutaneously. After 30 min, the animals were placed dorsally on the device with foot and knee secured. The tibialis anterior (TA) was stimulated subcutaneously with 0.1 ms supramaximal square-wave pulses at increasing frequencies (10, 20, 30, 40, 50, 60, 80, 100, 120, 140 Hz) to establish the force-frequency relationship. Maximal isometric tetanic force was determined by plateauing the force-frequency relationship. The maximal force was corrected by the TA mass. Next, TA muscle fatigue was assessed by repeated tetanic contractions of 0.1 ms duration and 120 Hz frequency. A total of 20 contractions were performed and the force maintenance index was calculated as the last contraction value, expressed as a percentage of the maximal force measured.

#### Spontaneous locomotion test

Spontaneous locomotion of R-DMDdup10-17 rats and their healthy wild-type littermates was assessed using a 6,000 cm^2^ observation roofless exercise cage, equipped with a camera filming from the top of the cage. This cage provided a spacious environment for spontaneous exploration, containing various features such as water and food dispensers, a shelter, an elevated mezzanine (mezza) and a ladder from the floor to the mezzanine. An automated artificial intelligence-based video monitoring software (EthoVision XT, Noldus, the Netherlands) was then used to assess rats’ locomotion and routine behavioral parameters. The system extracted metrics including the time spent in specific zones, the distance travelled, the average ground speed, and the number of successful climbs to the mezzanine (center point of the rat reaching the mezzanine).

#### Whole-body plethysmography

Respiratory capacities were evaluated using a whole-body plethysmograph (Emka Technology) on awake, unsedated animals. Each rat was placed in a calibrated cylindrical chamber at room temperature (RT). After 10 min of acclimatization, data were recorded for at least 15 min and analysed with the IOX software. Peak inspiratory (PIF) and expiratory (PEF) flow represented the maximal negative and positive recorded value.

#### Electrocardiography (ECG) analyses

Electrocardiograms were recorded on awake, unsedated rats using telemetry with skin patches and a battery-powered sensor worn in a jacket (rodent PACK, Emka Technologies). Two electrodes were placed on shaved and depilated skin, one dorsally in the cranial interscapular region and the other ventrally, in the region of the xiphoid appendix. The skin patches and vest were left on the animals for a 10-min habituation period into the rearing cage. Then, recording began for 10 to 20 min, corresponding to around 6,000 cardiac cycles. Data were analysed by the “Averaged beats analysis” plugin of the ECGavg software (v2), codeveloped with Emka [16]. To exclude the putative interference of the sympathetic nervous system hyperactivation during the acute phase of the stress response, we selected for each rat cycles between the lowest heart rate and up to 10% higher, which corresponded to the resting heart activity. The isoelectric line was defined as the TP segment; the end of the S wave was defined as the intersection between the trace and the isoelectric line, or in the case of a notched T wave (DMD rats), as the point of return to horizontal (J point). The corrected QTpeak value (QTpc) was calculated using the following modified Bazett’s formula: QTpc = QTpeak/(RR/f)1/2, derived from a previous study [17], with f equal to the mean RR interval calculated for each rat.

### Blood biomarkers

#### Plasma collection

All rats were anesthetised with the mixture described in the “In vivo muscle electrophysiology” section. After 30 min of induction, blood samples were taken from the retro-orbital sinus and collected in tubes containing lithium heparinate. Plasma was obtained by centrifuging tubes at 3000 rcf for 15 min at 4 °C, and was then aliquoted for immediate use or storage at -80 °C.

#### Quantification of plasma biomarkers (CK, MYOM3 and hs-cTnT)

CK plasma concentration was obtained with the Catalyst DX analyzer (IDEXX), following the manufacturer’s recommendations. If necessary, sera were diluted in NaCl, first 1/3, then 1/6, and 1/10 for the highest values. MYOM3 plasma concentration was obtained with the SimpleProtein Jess analyzer (BioTechne) using the regular 12–230 kDa kit, the polyclonal anti-MYOM3 antibody (ProteinTech #17692-1-AP, 1:2000) and the anti-rabbit HRP (ProteinSimple). Plasma were loaded at dilution 1:50 and the separation voltage was 375 V. All MYOM3 signal intensities were normalised to the individual total protein load, which was determined via Total Protein RePlex assay as per the manufacturer’s instructions. The high-sensitivity cardiac troponin T (hs-cTnT) plasma concentration was obtained with the hs-cTnT assay run on a Roche cobas 8000 analyser^®^ E801, following the manufacturer’s recommendations.

### Histological analyses

#### Muscle collection, freezing and storage

After blood collection, anaesthetised rats were then killed by exposure to carbon dioxide on a standardised cycle of 3 min to reach 40%, then a further 2 min to 70% carbon dioxide and a 10-min holding period in a 48 L CO_2_ chamber (Minerve). Muscles (*Tibialis anterior*, *Extensor digitorum longus*, *Gastrocnemius*, *Soleus*, diaphragm and heart) were dissected and harvested for histology. Each muscle was sealed in Tragacanth gum (Sigma Adrich) placed on a piece of cork and frozen in isopentane maintained at -140 °C by liquid nitrogen before being stored in a freezer at -80 °C until use. Transverse cryosections were obtained using a cryostat (Leica) and used for histological analysis and immunofluorescence.

#### Haematoxylin-eosin staining and pathological index quantification

Cryosections were stained with 0.1% Mayers hematoxylin (Sigma Aldrich) for 10 min, then counterstained with 0.5% eosin (Sigma Aldrich). After washing with distilled water, the sections were dehydrated in 50%, 70%, 95% and then 100% ethanol, before being incubated in xylene and mounted in Canada balsam. Photographs were taken using a ZEISS Axioscan 7 microscope, and images were analysed by Visilog (V6.4, Noesis). The pathological index was calculated as the proportion of events not corresponding to normal shape fibres as previously described [18].

#### Sirius red staining and fibrosis quantification

Frozen tissue sections were fixed for 30 min in 0.4% PFA. After washing with distilled water, sections were incubated with Sirius Red (Sigma Aldrich) for 10 min and counterstained in fast green for 4 min at RT. After a second wash with distilled water, the sections were dehydrated in 50%, 70%, 95% and then 100% ethanol, before being incubated in xylene and mounted in Canada balsam. Photographs were taken using ZEISS Axioscan 7, and images were quantified by Visilog (V6.4, Noesis) as relative area of fibrosis.

#### Immunofluorescence labelling of frozen sections and imaging

Cryosections were dried for at least 30 min, then rapidly rehydrated in PBS before being blocked in 10% BSA (Bovine Serum Albumin) for 45 min at RT. Slides were then incubated with primary antibodies raised against laminin (Sigma Aldrich, L9393) and dystrophin (Leica, NCL-DYS1). All antibodies were diluted in 0.1% BSA and incubated overnight at 4 °C. Slides were then exposed to Alexa Fluor secondary antibodies for 1 h at RT, rinsed, stained with Hoechst (Sigma Aldrich), and mounted with Fluoromount-G Mounting Medium (Invitrogen). Images were acquired using a fluorescence microscope (ZEISS Axioscan 7).

### Data analysis

#### Correction of inter-individual variability caused by growth disparities

Because DMD is a dystrophic myopathy whose progression is accompanied by massive muscle atrophy, the classical correction of values for inter-individual variations in growth rate by the body weight (BW), which depends mainly on the total mass of striated muscles, cannot be applied. Instead, we used an optimised version of the methodology initially developed by Hagdorn *et al.* for cardiovascular disease, who proposed using the cubic value of tibia length (TL^3^) as a reference for bone growth [19]. This tibia length is cubed to comply with the mathematical principle that mass reflects a volume rather than a linear dimension. We applied this method to our data and first demonstrated in WT rats of our Sprague Dawley line that correction solely by TL³ yielded homogeneous (statistically unsignificant) TA muscle mass values, irrespective of individual growth (Fig. S1A). By contrast, correction by the total body mass or the uncubed tibia length failed to eliminate these discrepancies (Fig. S1A), underlining the robustness of a biologically and mathematically appropriate correction. The accuracy of this correction has also been extended by taking the example of the testis, an organ whose morphology is visibly unaffected by the dystrophic phenotype (Fig. S1B). The standard correction of testis mass by total body mass shows that it was abusively increased in DMD rats (Fig. S1C). In contrast, correction by the TL^3^ value abolished this over-correction and confirmed the absence of any significant difference between DMD rats and their WT littermates (Fig. S1D).

Thus, organ weight data or strength values were corrected on tibia length (TL^3^), using correction parameters that we had previously calculated in WT animals for each variable, allowing to calculate linear regression between TL^3^ and the corresponding variable. This normalization was then completed to obtain a post-correction of the scale that considered the growth of the animals in the longitudinal study. The final formula was the following:

$$\:Individual\:corrected\:variable=\:$$

$$\eqalign{ {{ individual\;raw\;variable } \over { - X - intercept\;of\;corrector\; + \;individual\;value\;of\;corrector }} \times \cr ( - X - intercept\;of\;corrector\; + \;subgroup\;median\;of\;corrector) \cr} $$

*X-intercept was calculated from linear regression between the corrector and the considered variable to correct. Corrector was set as TL*^*3*^. *Subgroup was defined by the different time points where animals had been analysed (i.e. at 6 months and 10 months). To keep the last argument a constant for the specified time point*,* the median was calculated for the pooled rats*,* whatever their status (WT or DMD).*

### Statistical analyses

All experiments were analysed using GraphPad Prism v9 or v10. Depending on the results of the Shapiro-Wilk test assessing the normality of the data distribution, we used parametric or non-parametric tests for comparisons. With exceptions specifically mentioned in Figure legends, all the non-parametric data were analysed using the Kruskal-Wallis test followed by Dunn’s post-hoc, and parametric data were analysed using an ordinary one-way ANOVA followed by Sidak’s post-hoc to specifically compare data obtained at 6 months (WT vs. DMD), 10 months (WT vs. DMD) and the DMD progression (10 months vs. 6 months). Graph data are presented as mean and standard deviation, with each point representing a single biological repeat. All significant differences are shown in the graph as two-tailed p-values, with critical alpha set at 0.05.

## Results

### Absence of dystrophin and premature death in R-DMDdup10-17 rats

**Fig. 1:**
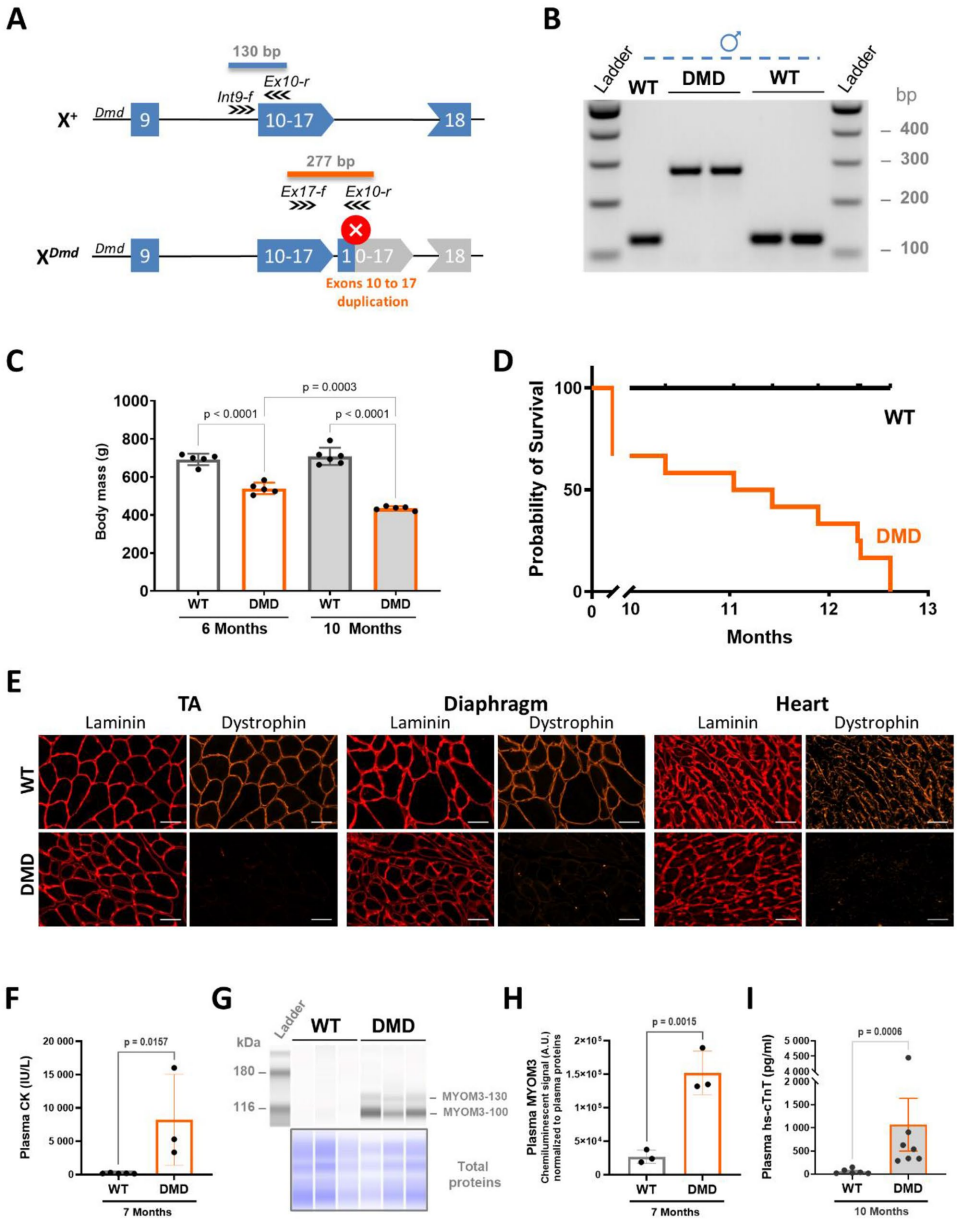
Early death associated with severe muscle atrophy in dystrophin-deficient R-DMDdup10-17 (DMD) rats. **(A)** Scheme of CRISPR/Cas9-mediated duplication on the rat chromosome X of a ~ 125 kb genomic region encompassing exons 10 to 17 of the *Dmd* gene. The wild-type allele (X^+^) is at the top, with the reading frame indicated by the blue colour of the exons. The allele containing the duplication (X^*DMD*^) is at the bottom. Splicing between exon 17 and the duplicated exon 10 induces a premature stop codon in the duplicated exon 10 (white cross on red background) and the absence of translation of subsequent exons (grey). The position and orientation of the primers used for genotyping is indicated by arrows, as is the expected amplicon size for each of the two alleles. **(B)** Electrophoretic gel of amplicons obtained using a multiplex of the three primers indicated in **(A)** from genomic DNA of male rats from a litter born from a carrier female and a WT male. A band at 130 bp reveals the WT allele, while a band at 277 bp reveals a male hemizygous for the *Dmd* allele carrying the duplicated 10–17 region. **(C)** Body mass of WT and DMD littermates at 6 and 10 months of age. **(D)** Kaplan-Meier curve for the frequency of WT (black curve) and DMD (orange curve) rat survival. **(E)** Representative immunofluorescence for laminin (red) and dystrophin (orange) at 6 months on TA, diaphragm and heart sections of WT and DMD littermates, showing complete absence of a dystrophin signal in DMD muscles. Scale bars, 50 μm. **(F)** Plasma CK levels in 7-month-old WT and DMD littermates. One-tailed unpaired t test. **(G)** Upper panel: Capillary immunoelectrophoresis plasma protein analysis detecting the two fragments (100 and 130 kDa) of the sarcomeric myomesin-3 (MYOM3) protein in 7-month-old DMD rats, but not in their WT littermates; lower panel: total proteins for each lane. **(H)** Quantification of plasma MYOM3 levels assessed in (**G**), corresponding to the sum of both MYOM3-130 and MYOM3-100 signals normalised to total plasma proteins. One-tailed unpaired t test. **(I)** Plasma hs-cTNT levels in 10-month-old WT and DMD littermates. One-tailed unpaired t test

Using the CRISPR/Cas9 genome editing method, we generated a new DMD rat model with a duplication of exons 10 to 17. This large ~ 125 bp genomic duplication is predicted to disrupt the coding frame of the full length Dp427 dystrophin, leading to a Stop codon in the duplicated exon 10 (Fig. 1A). A total number of 238 electroporated fertilised oocytes were reimplanted in foster females and an F_0_ founder male carrying the duplication was mated to generate the line that we have named R-DMDdup10-17, often abbreviated DMD in the present article. To minimize genetic drift and potential maternal effects on expression of the mutation, R-DMDdup10-17 male rats and wild-type (WT) controls were generated as littermates through breeding of carrier R-DMDdup10-17 females with WT Sprague Dawley males. The WT males were replaced twice a year using a controlled breeding stock (Janvier Laboratories). Hemizygous WT and R-DMDdup10-17 males were genotyped at 3 weeks of age (Fig. 1B), allowing a longitudinal follow-up of individuals. At 6 months of age, all DMD rats showed a significant decline in their body mass (BM) that worsened at 10 months (Fig. 1C). From 10 months onwards, DMD animals showed increasing difficulty in feeding themselves from the pellets provided in the cage grid. Initially, this difficulty was overcome by placing pellets on the floor, but this was later no longer sufficient, and in line with the limit points established for this line, we then euthanised the first animals at 10 months of age. Among a cohort of 12 R-DMDdup10-17 rats, the least affected survived until the age of 12 months (Fig. 1D).

Absence of dystrophin was validated by direct immunofluorescence on transverse sections of the tibialis anterior (TA) muscle, diaphragm and heart from 6-month-old DMD rats (Fig. 1E), with dystrophin-positive (revertant) fibres seldom observed. Blood levels of the conventional intracellular biomarker creatine kinase (CK) were markedly elevated in DMD rats relative to their healthy littermates (Fig. 1F), indicating widespread sarcolemma disruption. Selective necrosis of skeletal muscle fibres was further evidenced by a significant increase in circulating myomesin-3 (MYOM3) levels (Fig. 1G, H), while enhanced cardiomyocyte necrosis was reflected by elevated concentrations of high-sensitivity cardiac troponin T (hs-cTnT; Fig. 1I). Together, these biomarkers reveal ongoing, dystrophin-deficiency-driven myonecrosis in both skeletal and cardiac muscle.

In summary, duplication in rats of the genomic region containing exons 10 to 17 of the *Dmd* gene leads to a complete absence of dystrophin in skeletal and cardiac muscles, resulting in a progressive and severe weakness phenotype that leads to a shortened lifespan. This fatal disease trajectory parallels that observed in children carrying this type of mutation and is similar to a deletion 52 rat model in the same genetic background [14].

#### Generalised muscle atrophy in R-DMDdup10-17 rats

**Fig. 2:**
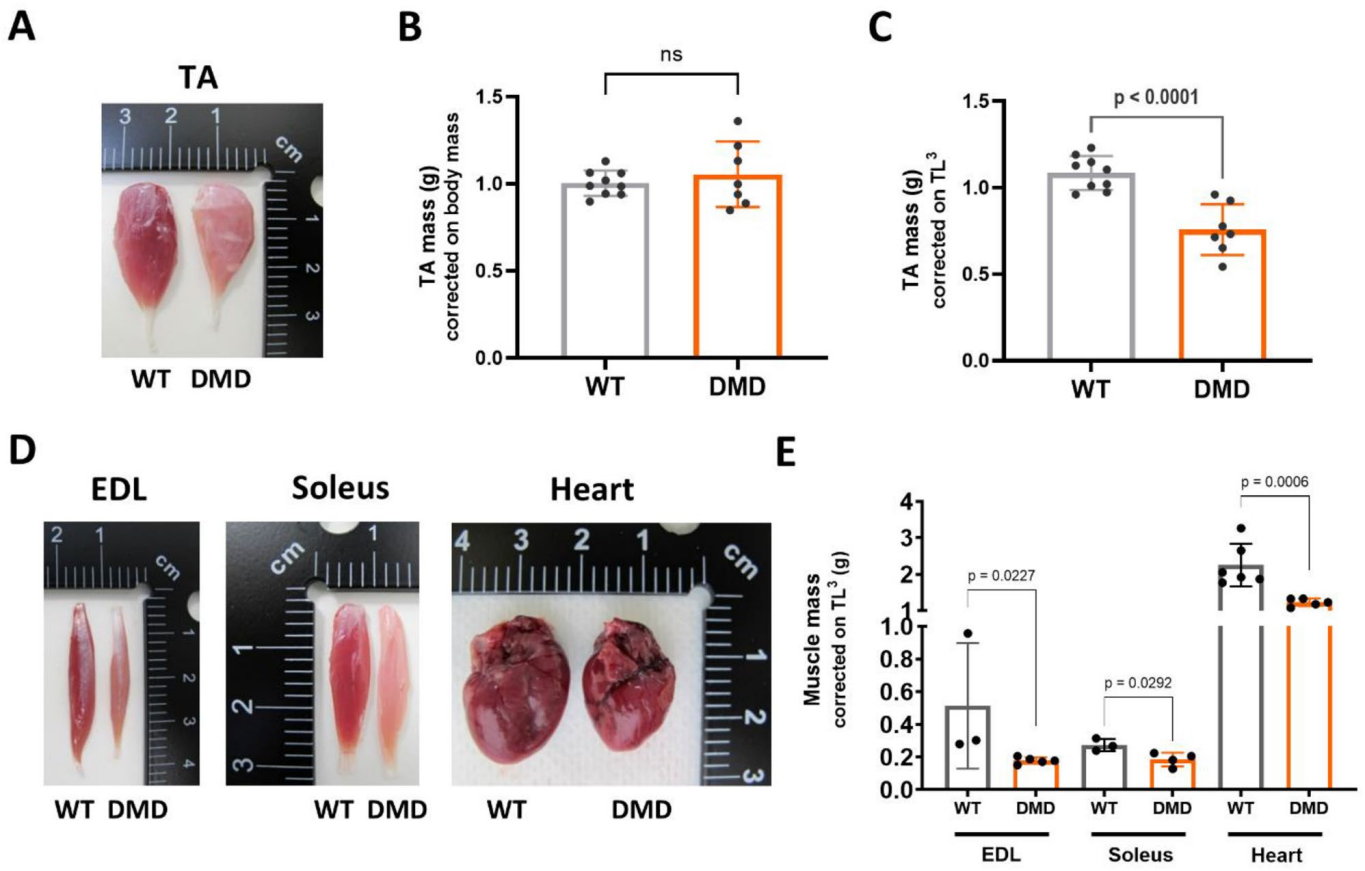
Global muscle atrophy in 10-mo-old R-DMDdup10-17 (DMD) rats. **A.** Picture of WT and DMD TA at 10 months. **B.** Mass of WT and DMD TA at 10 months, corrected on body mass (BM). Two-tailed unpaired t test. **C.** Mass of WT and DMD TA at 10 months, corrected on TL^3^. Two-tailed unpaired t test. **D.** Picture of WT and DMD EDL, soleus, and heart at 10 months. **E.** EDL, soleus, and heart at 10 months, corrected on TL^3^

At 10 months of age, the TA of a DMD rat was noticeably altered, appearing paler and markedly reduced in volume (Fig. 2A). Surprisingly, TA muscle mass was not significantly reduced in DMD rats compared to their healthy littermates when values were normalised with the total body mass of the rats, a method routinely used to correct for inter-individual variations in animal growth (Fig. 2B). The most likely explanation for this discrepancy between the image and the corrected mass value was an error in the choice of correction. Indeed, muscle mass represents on average 40% of the body mass of a mammalian organism. In DMD individuals, overall muscular atrophy therefore drastically reduces the value of body mass, which no longer reflects the individual’s growth, but instead its disease progression. The tibia length (TL) was therefore preferred to assess mild disparities in growth between individuals. Because a mass reflects a volumetric rather than a linear dimension, the cube tibia length (TL^3^) was eventually used to correct the TA muscle mass. We thus confirmed after correction that TA from 10-month-old DMD rats were significantly lighter than those of their WT littermates (Fig. 2C), in accordance with their reduced volume (Fig. 2A). This finding was also observed for the EDL, soleus and cardiac muscles collected at the same age. Their morphology suggested a reduced volume (Fig. 2D), and this was confirmed by a highly significant reduction in their corrected mass (Fig. 2E). At 6 months of age, we noted a pronounced trend towards reduced muscle mass, which was not statistically significant at the level of individual muscles, but highly significant when considering the cumulated muscle mass, represented by the body mass of the animals (Table 1).

**Table 1:** Whole body and individual muscle mass

Mass		6 Months			10 Months	
		WT	DMD		WT	DMD
Whole body (g)		692.0 ± 30.3	539.8 ± 30.0 ****		708.7 ± 45.7	435.0 ± 10.8 **** $$$
Tibialis anterior (g)		0.97 ± 0.14	0.89 ± 0.08		1.04 ± 0.07	0.80 ± 0.14 **
EDL (g)		0.26 ± 0.02	0.21 ± 0.03		0.51 ± 0.38	0.18 ± 0.02 *
Soleus (g)		0.28 ± 0.05	0.26 ± 0.02		0.27 ± 0.04	0.19 ± 0.04 * $
Heart (g)		2.1 ± 0.2	1.7 ± 0.3		2.3 ± 0.6	1.2 ± 0.1 **

*p* value calculated referred to WT (*) or DMD at 6 Months ($).

*/$ *p* < 0.05

** *p* < 0.01

$$$ *p* < 0.001

**** *p* < 0.0001

In summary, we confirmed that dystrophin deficiency in R-DMDdup10-17 rats leads to progressive muscle atrophy, particularly pronounced at 10 months of age.

### Histological and functional impairments in limb musculature

**Fig. 3:**
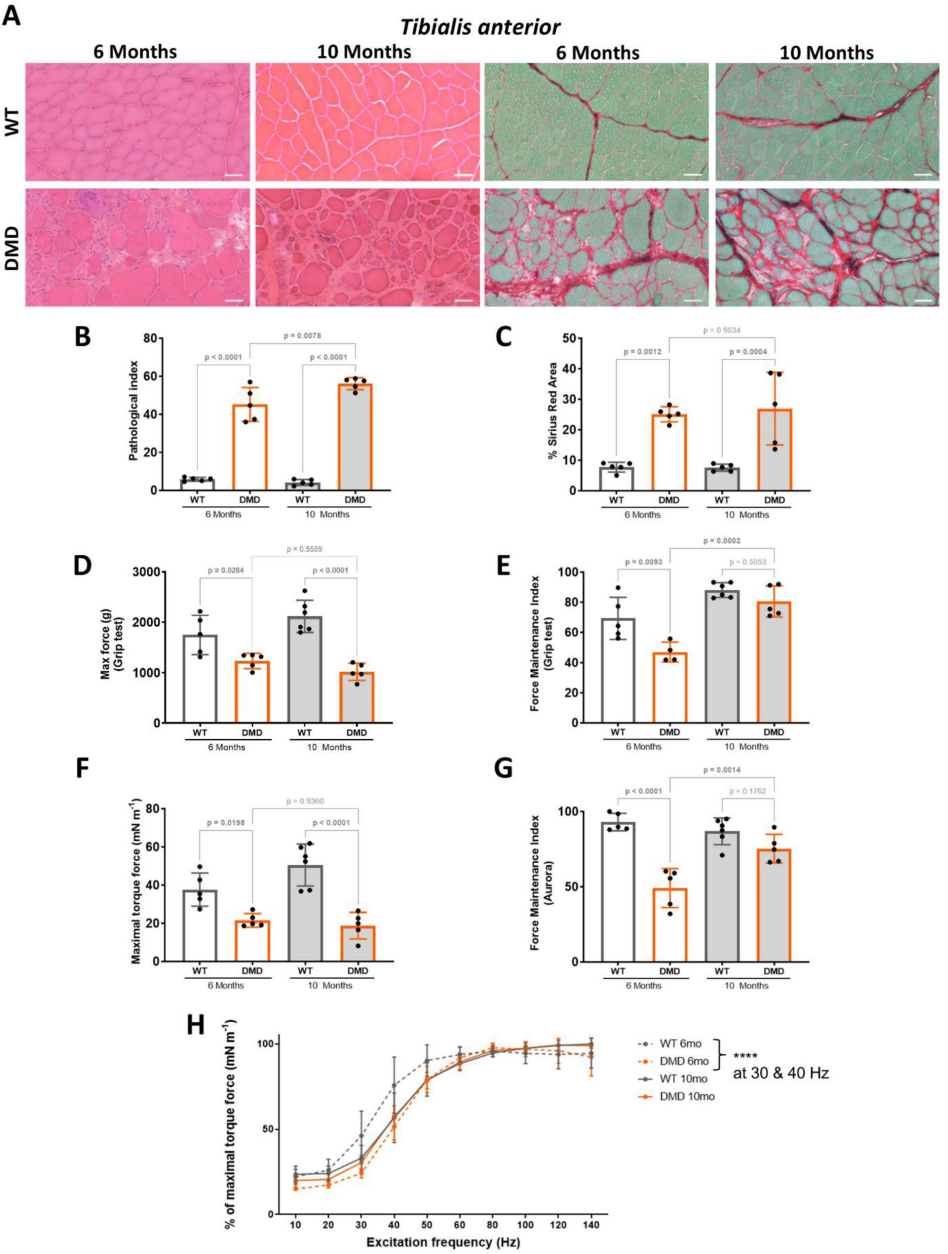
Histological and functional impairment of R-DMDdup10-17 (DMD) skeletal muscles. **A.** Haematoxylin and eosin (HE, left panels) and Sirius Red (SR, right panels) staining of the tibialis anterior (TA) from 6- and 10-month-old WT and DMD littermates. Scale bars, 50 μm. **B.** Pathological index calculated on HE-stained transverse sections of TA from 6- and 10-month-old WT and DMD littermates. **C.** Quantification of fibrotic deposition based on SR-stained area of transverse sections of TA from 6- and 10-month-old WT and DMD littermates. **D** and **E**. Corrected maximal force of forelimbs by grip test **(D)** and corrected force maintenance index **(E)** in 6- and 10-month-old WT and DMD littermates. **F** and **G**. Corrected maximal torque force **(F)** and force maintenance index **(G)** of the TA after direct muscle stimulation in 6- and 10-month-old WT and DMD littermates. **H**. Percentage of force-frequency relationship representative of excitation/contraction coupling ratio in 6- and 10-month-old WT and DMD littermates

To further assess the microstructure of the dystrophin-deficient TA muscle, we analysed cross-sections stained with either haematoxylin and eosin (HE) or Sirius red (SR). At both 6 and 10 months of age, we found fibre morphology alterations, associated with invading inflammatory cells and fibrotic deposition (Fig. 3A). At both ages, HE staining enabled to calculate a global pathological index that was significantly increased in DMD versus WT littermates, and also significantly increased at 10 months compared to 6 months (Fig. 3B). Similarly, SR staining at both 6 and 10 months of age revealed a significantly increased fibrotic deposition in DMD versus WT littermates, with no progression between 6 and 10 months (Fig. 3C).

To quantify the functional consequences of this prominent histological remodelling, we assessed muscle strength in rats. First, we measured the combined force of forelimb muscles using a grip test on conscious animals. Then, to ensure an accurate assessment of muscle strength, independent of potential variations in motivation during the grip test, and to better evaluate force of the muscle relative to its mass, we additionally measured the force generated by a single muscle, the TA, through its direct electrical stimulation in anesthetised animals. Of note, force correlates with muscle mass in normal conditions. Here again, we used the equidimensional TL^3^ correction method for all force data. Using the grip test, we quantified a significantly reduced maximal force in DMD rats compared to their WT littermates, at 6 and 10 months of age (Fig. 3D). The force maintenance index, representative of muscle fatigue during grip repetitive tests, was also significantly reduced at 6 months (Fig. 3E). Direct stimulation of the TA muscle under balanced anaesthesia confirmed a significant reduction in overall relative force at 6 and 10 months (Fig. 3F), and in the force maintenance index at 6 months in DMD rats, compared to their WT littermates (Fig. 3G). Analysis of the force-frequency relationship showed a right shift of the curve in all DMD and 10-month-old WT rats, compared to younger 6-month-old WT rats (Fig. 3H). This suggested an early, non-progressive impairment in the excitation-contraction coupling of DMD rats.

#### Reduced spontaneous locomotion in R-DMDdup10-17 rats

**Fig. 4:**
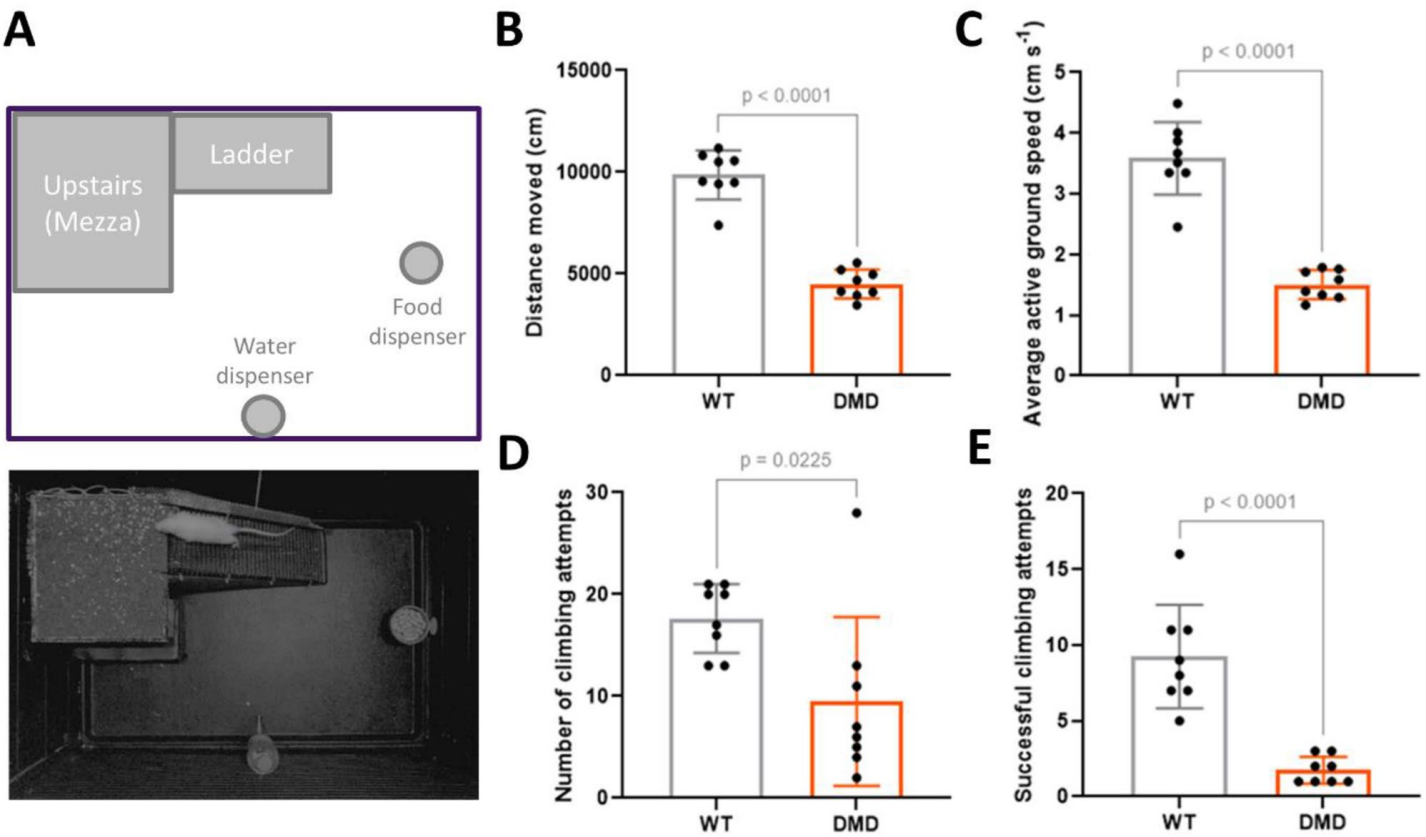
Reduced spontaneous locomotion in 6-month-old R-DMDdup10-17 (DMD) rats. **A**. Scheme (upper panel) and picture (lower panel) of the large exercise arena equipped with a mezzanine (Mezza) and a ladder connecting the mezza to the ground. **B-E**. Total distance moved (**B**), average of the active ground speed (**C**), total number of climbing attempts (**D**), and total number of successful climbing (reaching the mezzanine, **E**) calculated in video-tracked 6-month-old WT and DMD littermates. Two-tailed unpaired t tests

To fully assess the impact of functional muscle deficits while minimising interference from human experimenters, we placed the rats individually in an exercise cage and filmed them for 45 min (Fig. 4A). Post hoc analysis of the recordings enabled us to quantify their spontaneous locomotion parameters. At 6 months of age, the analysis revealed a highly significant reduction in the total distance travelled by DMD rats compared with their WT littermates, who travelled twice as far over the same period (Fig. 4B). This reduction was associated in DMD rats with a halving of their running speed (Fig. 4C), which alone explains the reduction in the distance travelled. In addition, DMD rats showed poorer overall performance when stepping onto the ladder connecting the mezzanine to the ground (Fig. 4D), although inter-individual variability resulted in a marginally significant difference (*p* = 0.02). More notably, DMD rats uniformly failed to reach the mezzanine (Fig. 4E), frequently turning back during most of their attempts to climb the ladder.

#### Progression of histological remodelling of the diaphragm and ventilatory defects in R-DMDdup10-17 rats

**Fig. 5:**
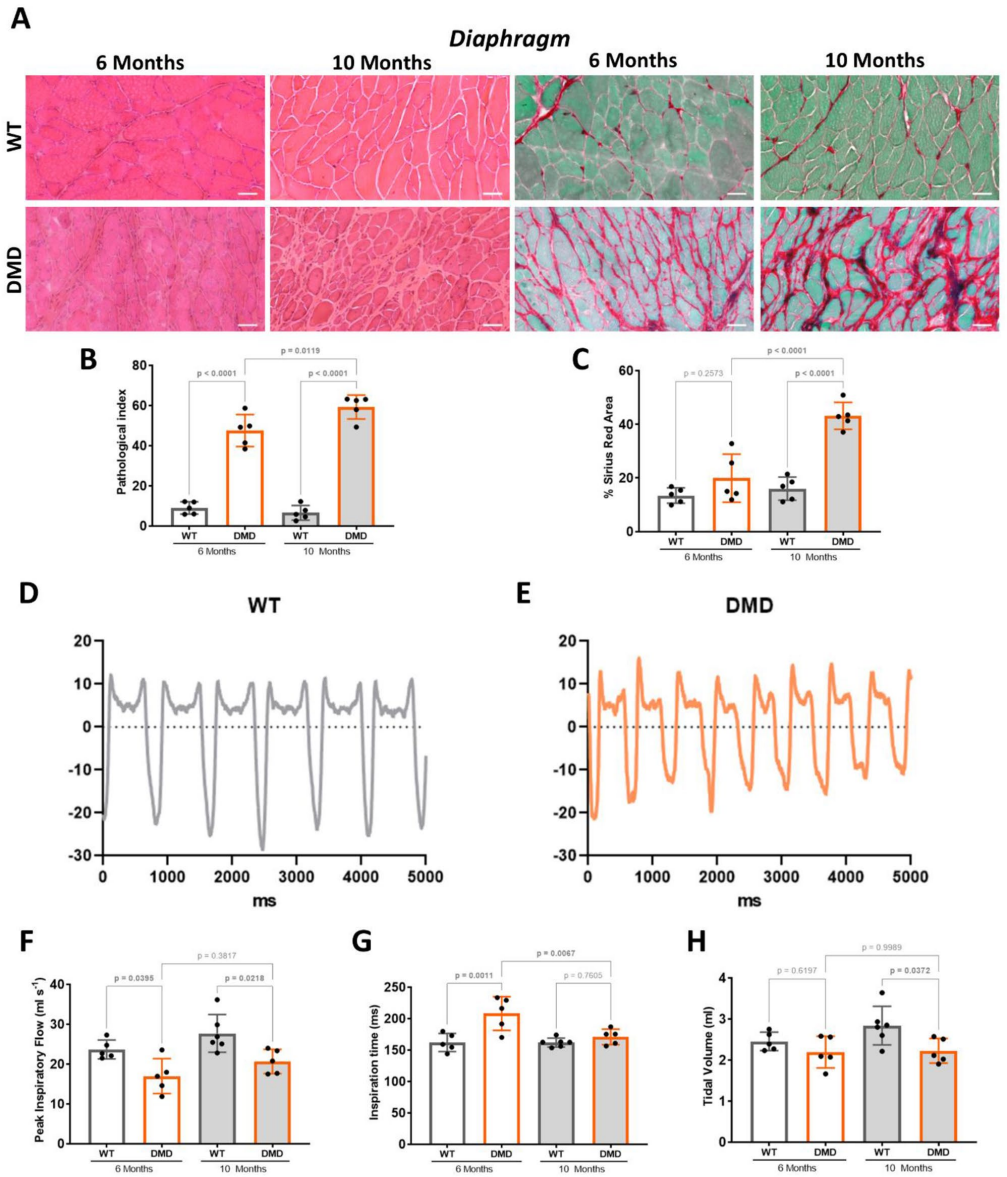
Histological and functional impairment of ventilatory muscles from R-DMDdup10-17 (DMD) rats. **A.** Haematoxylin and eosin (HE, left panels) and Sirius Red (SR, right panels) staining of diaphragm from 6- and 10-month-old WT and DMD littermates. Scale bars, 50 μm. **B.** Pathological index calculated on HE-stained transverse sections of diaphragm from 6- and 10-month-old WT and DMD littermates. **C.** Quantification of fibrotic deposition based on SR-stained area of transverse sections of diaphragm from 6- and 10-month-old WT and DMD littermates. **D-E**. Representative traces of ventilatory cycles in WT **(D)** and DMD **(E)** littermates at 6 months. Negative flow peaks indicate depression in the box, i.e. the inspiration step for the animal, while the positive peaks reflect the expiration. **F-H**. Quantification of peak inspiratory flow **(F)**, inspiration time **(G)**, and tidal volume **(H)** measured by whole-body plethysmography in 6-month-old WT and DMD littermates

We observed cross-sections of the diaphragm stained with both haematoxylin and eosin (HE) and Sirius red (SR). At both 6 and 10 months of age, we found fibre morphology alterations, associated with invading inflammatory cells and fibrotic deposition (Fig. 5A). At both ages, the global pathological index was significantly increased in DMD versus WT littermates, and in addition it was significantly increased at 10 months compared to 6 months (Fig. 5B). The fibrotic deposition was significantly and progressively increased in DMD versus WT littermates at 6 and 10 months of age (Fig. 5C).

To correlate this histological remodelling with ventilatory function at rest, which is mostly diaphragm-dependent, we performed a non-invasive whole-body plethysmography evaluation in conscious inactive rats. Representative traces obtained in 6-month-old rats showed modified resting ventilatory cycles in DMD rats (Fig. 5D and E), characterised by a significantly reduced peak inspiratory flow (Fig. 5F) and a compensatory increased inspiration time (Fig. 5G), ultimately preserving the tidal volume (Fig. 5H), compared to their WT littermates. At 10 months, the reduction in peak inspiratory flow was no longer compensated during this evaluation, resulting in a significant decrease in the tidal volume (Fig. 5F-H).

#### Progressive cardiac remodelling and electrocardiographic defect in R-DMDdup10-17 rats

**Fig. 6:**
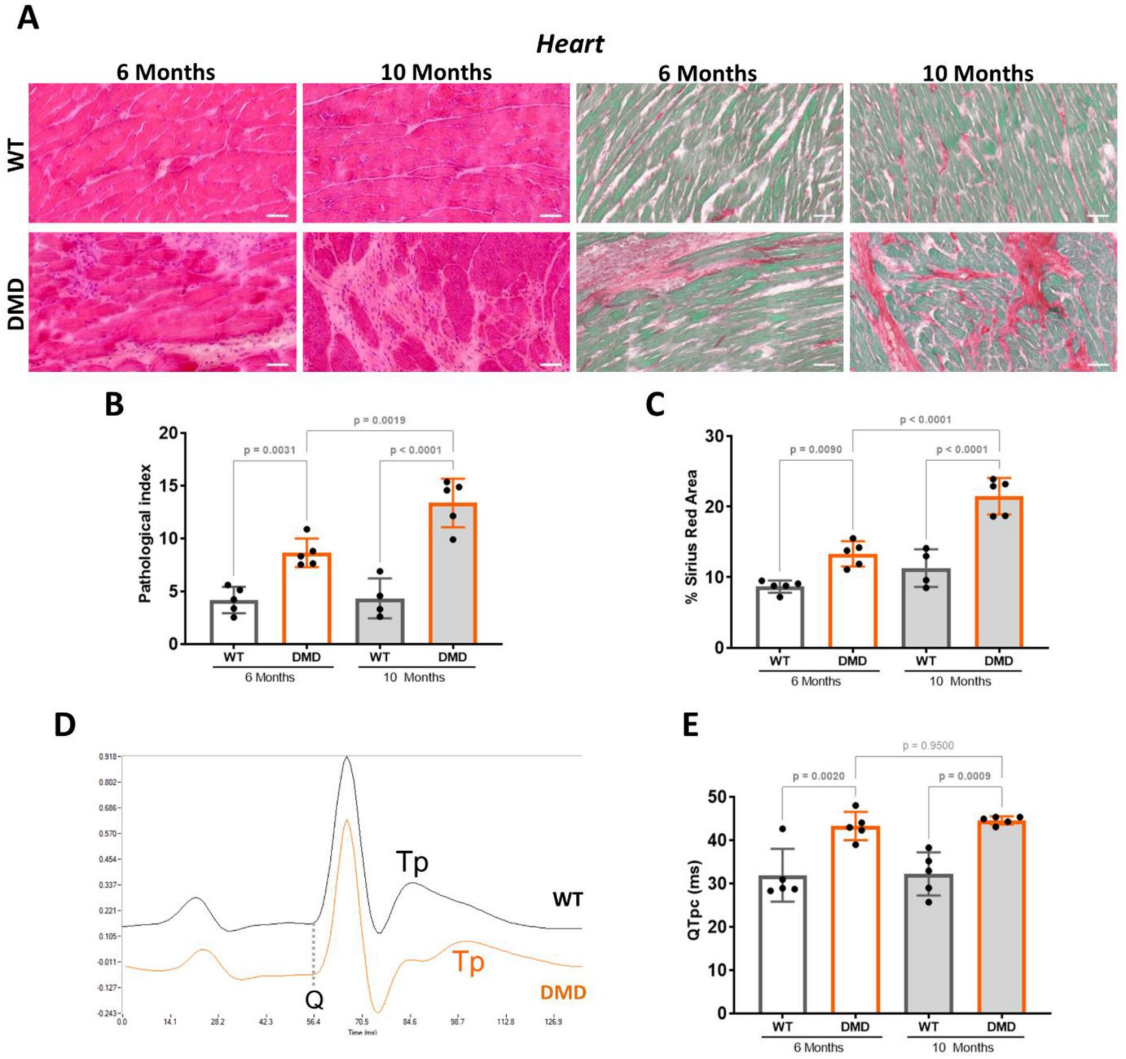
Histological and functional impairment of the heart from R-DMDdup10-17 (DMD) rats. **A.** Haematoxylin and eosin (HE, left panels) and Sirius Red (SR, right panels) staining of the left ventricle free wall of the heart from 6- and 10-month-old WT and DMD littermates. Scale bars, 50 μm. **B.** Pathological index calculated on HE-stained transverse sections of the whole heart from 6- and 10-month-old WT and DMD littermates. **C.** Quantification of fibrotic deposition based on SR-stained area of transverse sections of the whole heart from 6- and 10-month-old WT and DMD littermates. **D.** Representative averaged ECG trace of 6-month-old WT (grey) and DMD (orange) littermates. Q represents the onset of ventricles depolarization. Tp represents the peak of the T wave (repolarization of the ventricles), and QTp is the time between Q and Tp. **E.** Quantification of the heart rate-corrected QTp (QTpc) interval in 6- and 10-month-old WT and DMD littermates

We observed cross-sections of the heart stained with both haematoxylin and eosin (HE) and Sirius red (SR). Representative pictures taken at the level of the free wall of the left ventricle are shown (Fig. 6A). At 6 and 10 months of age, the global pathological index was significantly increased in DMD versus WT littermates, and in addition it was significantly increased at 10 months compared to 6 months (Fig. 6B). At 6 months of age, the fibrotic deposition was also significantly increased in DMD versus WT littermates, and it was exacerbated at 10 months (Fig. 6C).

We have previously demonstrated that the resting ECG of R-DMDdel52 rats is characterised by a prolonged QTpc interval [14], a marker of the time from the onset of ventricular depolarisation to the peak of the T wave, which reflects ventricular repolarisation. The highly significant, non-progressive increase in QTpc observed in Del52 rats is associated with histological features very similar to those reported here in R-DMDdup10-17 rats. Therefore, we performed ECG recordings in R-DMDdup10-17 rats, using a non-invasive approach minimising the contribution of excessive sympathetic innervation typically activated during a stress response. Indeed, at both 6 and 10 months, the heart rate of DMD rats was not significantly different from that of their WT littermates (6 months: DMD = 421 ± 48 bpm, WT = 454 ± 47 bpm and 10 months: DMD = 421 ± 41 bpm, WT = 409 ± 40 bpm; *p* = 0.400, One-way ANOVA test). In these conditions, all animals displayed a regular sinus rhythm, regardless of their genotype or age. Analysis of ECG traces revealed a notched T wave in DMD, but not WT rats (Fig. 6D), resulting in a significant, non-progressive increase in the QTpc interval (Fig. 6E).

Altogether, these data demonstrated that dystrophin deficiency in both R-DMDdel52 [14] and R-DMDdup10-17 Sprague Dawley rats induces similar myonecrosis-driven cardiac remodelling and a highly specific notched T wave leading to an increase of the QTpc biomarker.

## Discussion

In this work, we report the first severe animal model of a dystrophin deficiency linked to a duplication in the 5’ region of the *Dmd* gene, encompassing exons 10 to 17, and established a line that we named R-DMDdup10-17. By removing the mechanical protection conferred by the membrane complex normally organised around dystrophin, the duplication resulted in myonecrosis induced by membrane damage in the skeletal and heart muscles, as evidenced by the high blood levels of the two released biomarkers, MYOM3 and hs-cTnT. The concomitant signature of long-lasting necrosis was visible on histological sections of all the striated muscles analysed, juxtaposing an infiltration of inflammatory cells, interstitial fibrosis and the presence of small-diameter fibres with centralised nuclei, together defining a significantly elevated pathological index in DMD rats. Replacement of myofibrils by non-contractile, connective and adipose tissues resulted in loss of muscle force and mass that led to a reduction in body mass already significant at 6 months of age. It is very likely that muscle weakness occurs earlier than 6 months, as previously demonstrated in the R-DMDdel52 model [14]. Further evaluation of the Dup10-17 model at younger ages would provide a more comprehensive description of disease onset.

Because animal models are used for translational purposes and all animal research must comply with the principle of ethical refinement, we are constantly striving to develop the least invasive functional evaluation tests. Here, we combined the quantification of spontaneous locomotion parameters, ventilatory capacity, and cardiac electrical activity in awake, non-sedated rats to unambiguously reveal the functional deficits in DMD rats compared to their healthy WT littermates. First, the reduced spontaneous locomotion speed and inability of DMD rats to fully ascend a ladder in an exercise arena correlated with the reduced relative muscle force determined through direct invasive stimulation of the single TA muscle. It also preceded the significant reduction in individual muscle mass. Second, the reduced capacity of DMD resting rats placed in a whole-body plethysmography chamber to generate maximal inspiratory flow equivalent to WT rats correlated with the histological abnormalities observed post-mortem in diaphragms of animals. Third, the presence in DMD rats of a notched T-wave detectable via non-invasive telemetric ECG, and quantified through the QTpc parameter, correlated with the cardiac remodelling identified through post-mortem histological analysis of the heart. Altogether, these non-invasive tools provide a reliable set of biomarkers, readily applicable for longitudinal studies and the evaluation of candidate therapies.

Regarding limb muscle force, we observed a significant reduction in the force maintenance index at 6 months in DMD rats compared with their healthy littermates, measured by grip tests and direct stimulation of the TA muscle. This reduction was not observed at 10 months, which seems to contradict the progressive muscle deterioration. The force maintenance index is expressed as a percentage of maximal force, the value of which was not reduced between 6 and 10 months in DMD rats. We thus propose that this improved ability of muscle fibres to maintain constant force at 10 months may result from elevated intracellular concentrations of calcium ions during the experiment, potentially due to influx across the sarcolemma or to leakage through the ryanodine receptors, as previously documented in DMD conditions [20–22]. This hypothesis, among other possibilities, should be further explored.

At 6 months, the reduction in peak inspiratory flow was associated with an increased inspiration time, a compensatory mechanism. Of note, this functional compensation was not observed at 10 months in our experimental conditions. One explanation could be an increased difficulty caused by the progressive fibrosis. These results align with the respiratory impairments observed in DMD patients, where the progressive decline in tidal volume is a hallmark of disease progression. In humans, this reduction is primarily driven by diaphragm dysfunction, amplified by weakening and damage to the accessory skeletal muscles of the abdomen and thorax that support ventilation [23].

ECG variations were observed in R-DMDdup10-17 rats, similarly to DMD human patients [24–27]. As previously pointed out [14], ECG abnormalities are easy and inexpensive to detect, and they can precede myocardial functional decline [28]. They can also provide predictive markers of heart failure, to be used in preclinical evaluation of innovative therapies. Both R-DMDdup10-17 and R-DMDdel52 rat models appear to be highly relevant model for studying the histological and functional cardiac changes associated with DMD. Indeed, the abnormal notched T wave and the associated increased QTpc parameter are easy to record and calculate, and they proved to be a specific readout of dystrophin-deficient rats [14, 16]. The notched T wave resulting in a prolonged QTpc combined with normal P waves highlights a defect in the activation-repolarization cycle of the ventricles, likely resulting from disorganised intercalated discs [16, 29]. Indeed, disorganisation of cardiomyocyte junctions likely contributes to the early impaired electrical synchronization and cardiac subclinical dysfunction, possibly later amplified by progression of fibrosis. To further confirm the severe cardiac phenotype and monitor disease progression, future studies should include mechanical assessment of cardiac function–such as echocardiographic evaluation of ventricular dilation and ejection fraction.

Overall, this new DMD model confirmed that rat models lie between the mildly affected mdx mice [7, 30] and large animal models such as dogs, pigs and monkeys [6, 8, 9]. Indeed, the R-DMDdup10-17 rat model closely resembles our previous R-DMDdel52 model, both paralleling the human disease trajectory. Rat models of severe DMD offer a more accessible and cost-effective approach compared to pigs, dogs, or monkeys for investigating the cellular and molecular mechanisms of the disease that remain poorly understood. Moreover, the duplication mutation described here is a unique opportunity to explore innovative targeted CRISPR-based gene therapies, which already proved feasible [31, 32]. Because the R-DMDdup10-17 and R-DMDdel52 rat lines were generated and are maintained in the outbred Sprague Dawley genetic background [19], inter-individual variability is more pronounced than in an inbred mouse line. This provides greater variability in individual response to the innovative treatments being tested in preclinical trials, which is a genuine added value of these models. Hence, a single outbred DMD rat line offers benefits in translational myology and may recapitulate the use of distinct inbred mdx mouse, whose genetic background greatly influences the dystrophic phenotype [14].

Due to the Sprague Dawley genetic heterogeneity, WT and DMD rats within our lines exhibit size variations at a given age. Typically, muscle mass variability amongst individuals is adjusted by normalising against body mass, which serves as an indicator of overall growth. This approach assumes that a lighter individual is smaller in size, necessitating an upward adjustment of its organ mass for comparison with other, heavier rats. However, our findings demonstrate that correction of biological biomarkers (including muscle mass and strength) based on body mass is potentially misleading in DMD rats. In this case, the reduced body mass resulting from atrophy is erroneously interpreted as a growth delay, leading to inflated corrected values. To address this correction bias, we propose utilising tibia length as an alternative normalisation factor, as it remains independent of muscle and body mass. This method has proven robust and may be applicable to various clinical scenarios associated with mass loss independent of growth, such as cachexia, sarcopenia, or any muscular atrophy condition.

Combined R-DMDdup10-17 and R-DMDdel52 rat models also offer possibilities to work, in the same genetic background, on functions that, in systems outside the striated musculature, rely on specific dystrophin isoforms. Indeed, because transcription of the RNA encoding Dp140 starts from a promoter in intron 44, Dp140 expression should be unchanged in R-DMDdup10-17 rats, whereas R-DMDdel52 rats probably do not express Dp140. Both models therefore fail to express Dp427 but should differ in Dp140 expression. Dp140 has been shown to play a crucial role in neurodevelopment and cognitive functions such as learning, memory, and social behaviour [33–35]. By comparing these models, we can gain important insights into the molecular mechanisms disrupted by each dystrophin isoform expressed in the nervous system, highlighting their contribution to neurologic disorders in DMD and identifying mutation-specific therapeutic strategies for cognitive defects. Ongoing investigations using these complementary models will be presented in a forthcoming dedicated report.

## Conclusions

Duplication of the *Dmd* genomic region spanning exons 10 to 17 in the rat causes complete dystrophin deficiency, severe striated muscle dystrophy and premature death, establishing the R-DMDdup10-17 line as a unique mammalian model of a severe, lethal duplication variant in the gene encoding dystrophin. This study confirms the potential of the R-DMDdup10-17 line as a tool for the preclinical evaluation of targeted gene therapies for such mutations. By enabling comparisons with models of other types of mutations, it provides the opportunity to deepen our understanding of the pathogenic mechanisms specific to each class of mutations, thereby elucidating the origins of the distinct multisystemic deficiencies observed in different groups of patients eligible for precision therapies, based on the unveiled specific mechanisms.

## Electronic supplementary material

Below is the link to the electronic supplementary material.

Supplementary Material 1: Fig. S1: Inter-individual body mass correction bias in the case of muscular atrophy. **A.** Scatter plot illustrating the effect of correction for inter-individual growth disparities in TA mass by the body mass (left), the tibia length (centre) and the cube of the tibia length (right) for a group of sixty WT rats split into two groups around the body mass median (the 50% lightest and the 50% heaviest WT rats). Only correction by the cubic value of the tibia length efficiently corrects the mass of the TA according to the difference in growth between the heaviest and lightest rats. **B.** Representative picture of testes from 9-month-old WT (left) and DMD (right) littermates, showing no visible morphological difference between the two. **C.** Quantification of WT and DMD testes mass at 9 months, corrected on body mass (BM). Two-tailed unpaired t test. **D.** Quantification of WT and DMD testes mass at 9 months, corrected on TL^3^, supporting the absence of mass difference in accordance with (B). Two-tailed unpaired t test

## Data Availability

No datasets were generated or analysed during the current study.

## Abbreviations

BM: Body mass
CK: Creatine kinase
DAGC: Dystrophin-associated glycoprotein complex
DMD: Duchenne muscular dystrophy
EDL: Extensor digitorum longus
Ex: Exon
FMI: Force maintenance index
gRNA: Guide RNA
hs-cTnT: High-sensitivity cardiac troponin T
MYOM3: Myomesin-3
PEF: Peak expiratory flow
PIF: Peak inspiratory flow
R-DMD: Rat model of DMD
TA: Tibialis anterior
TL: Tibia length
WT: Wild-type
