# Supplementary material for: Extensive striated muscle damage in a rat model of Duchenne muscular dystrophy with Dmd exons 10–17 duplication
Source: Skelet Muscle. 2025 Jun 9;15:16. doi: 10.1186/s13395-025-00386-2 (PMC12147255; doi:10.1186/s13395-025-00386-2)
Supplement: Supplementary file 1 — Supplementary Material 1: Fig. S1: Inter-individual body mass correction bias in the case of muscular atrophy. A. Scatter plot illustrating the effect of correction for inter-individual growth disparities in TA mass by the body mass (left), the tibia length (centre) and the cube of the tibia length (right) for a group of sixty WT rats split into two groups around the body mass median (the 50% lightest and the 50% heaviest WT rats). Only correction by the cubic value of the tibia length efficiently corrects the mass of the TA according to the difference in growth between the heaviest and lightest rats. B. Representative picture of testes from 9-month-old WT (left) and DMD (right) littermates, showing no visible morphological difference between the two. C. Quantification of WT and DMD testes mass at 9 months, corrected on body mass (BM). Two-tailed unpaired t test. D. Quantification of WT and DMD testes mass at 9 months, corrected on TL3, supporting the absence of mass difference in accordance with (B). Two-tailed unpaired t test [file 13395_2025_386_MOESM1_ESM.docx]

**Supplementals**


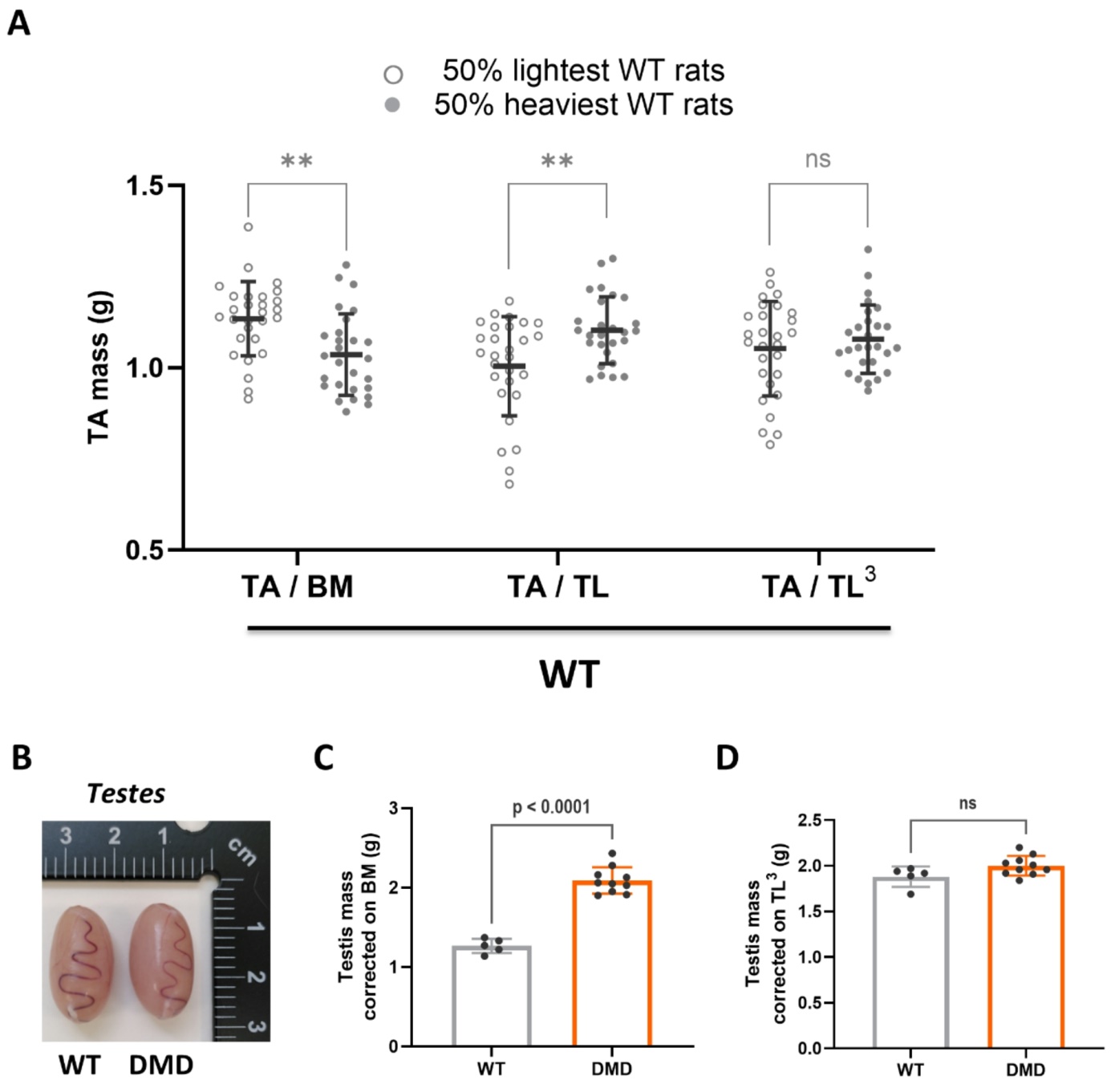


**Fig. S1:** Inter-individual body mass correction bias in the case of muscular atrophy. **A.** Scatter plot illustrating the effect of correction for inter-individual growth disparities in TA mass by the body mass (left), the tibia length (centre) and the cube of the tibia length (right) for a group of sixty WT rats split into two groups around the body mass median (the 50% lightest and the 50% heaviest WT rats). Only correction by the cubic value of the tibia length efficiently corrects the mass of the TA according to the difference in growth between the heaviest and lightest rats. **B.** Representative picture of testes from 9-month-old WT (left) and DMD (right) littermates, showing no visible morphological difference between the two. **C.** Quantification of WT and DMD testes mass at 9 months, corrected on body mass (BM). Two-tailed unpaired t test. **D.** Quantification of WT and DMD testes mass at 9 months, corrected on TL^3^, supporting the absence of mass difference in accordance with (B). Two-tailed unpaired t test.
